# Senescent thyrocytes and thyroid tumor cells induce M2-like macrophage polarization of human monocytes via a PGE2-dependent mechanism

**DOI:** 10.1186/s13046-019-1198-8

**Published:** 2019-05-21

**Authors:** Mara Mazzoni, Giuseppe Mauro, Marco Erreni, Paola Romeo, Emanuela Minna, Maria Grazia Vizioli, Cristina Belgiovine, Maria Grazia Rizzetti, Sonia Pagliardini, Roberta Avigni, Maria Chiara Anania, Paola Allavena, Maria Grazia Borrello, Angela Greco

**Affiliations:** 1 Molecular Mechanisms Unit, Department of Research, Fondazione IRCCS Istituto Nazionale dei Tumori, Via G.A. Amadeo, 42, 20133 Milan, Italy; 2 Department of Immunology, IRCCS Humanitas Clinical and Research Center, Via Manzoni, 56, 20089 Rozzano Milan, Italy; 3 Beatson Institute for Cancer Research, Bearsden, Glasgow, UK; 4 Institute of Cancer Sciences College of Medical, Veterinary and Life Sciences, University of Glasgow, Glasgow, G61 1BD UK

**Keywords:** Thyroid carcinoma, HRAS, Thyrocytes, Oncogene-induced senescence, Macrophages, COX-2, PGE2

## Abstract

**Background:**

Thyroid carcinoma includes several variants characterized by different biological and clinical features: from indolent microcarcinoma to undifferentiated and aggressive anaplastic carcinoma. Inflammation plays a critical role in thyroid tumors. Conditions predisposing to cancer, as well as oncogene activity, contribute to the construction of an inflammatory microenvironment that facilitates thyroid tumor progression. Moreover, oncogene-induced senescence, a mechanism tightly connected with inflammation, and able to restrain or promote cancer progression, is involved in thyroid cancer. The interactions between thyroid tumor cells and the microenvironment are not completely clarified.

**Methods:**

We characterize in vitro the interplay between macrophages and senescent thyrocytes and tumor-derived cell lines, modeling early and late thyroid tumor stages, respectively. Purified peripheral blood-derived human monocytes were exposed to thyroid cell-derived conditioned medium (CM) and assessed for phenotype by flow cytometry. The factors secreted by thyroid cells and macrophages were identified by gene expression analysis and ELISA. The protumoral effect of macrophages was assessed by wound healing assay on K1 thyroid tumor cells. The expression of PTGS2 and M2 markers in thyroid tumors was investigated in publicly available datasets.

**Results:**

Human monocytes exposed to CM from senescent thyrocytes and thyroid tumor cell lines undergo M2-like polarization, showing high CD206 and low MHC II markers, and upregulation of CCL17 secretion. The obtained M2-like macrophages displayed tumor-promoting activity. Among genes overexpressed in polarizing cells, we identified the prostaglandin-endoperoxide synthase enzyme (PTGS2/COX-2), which is involved in the production of prostaglandin E2 (PGE2). By using COX-2 inhibitors we demonstrated that the M2-like polarization ability of thyroid cells is related to the production of PGE2. Co-expression of PTGS2 and M2 markers is observed a significant fraction of human thyroid tumors.

**Conclusions:**

Our results demonstrate that both senescent thyrocytes and thyroid tumor cell lines trigger M2-like macrophage polarization that is related to PGE2 secretion. This suggests that the interaction with the microenvironment occurs at both early and late thyroid tumor stages, and favors tumor progression. The co-expression of PTGS2 gene and M2 markers in human thyroid carcinoma highlights the possibility to counteract tumor growth through COX-2 inhibition.

**Electronic supplementary material:**

The online version of this article (10.1186/s13046-019-1198-8) contains supplementary material, which is available to authorized users.

## Introduction

Thyroid carcinoma is the most frequent endocrine neoplasia. The majority of thyroid tumors arise from follicular thyroid cells and include papillary (PTC), follicular (FTC), poorly differentiated (PDTC), and anaplastic (ATC) carcinoma. PTC is the most common histotype (80%) and includes several histological variants characterized by different clinical outcomes: from papillary thyroid microcarcinoma (PTMC), considered the early stage of PTC and with favorable prognosis, to tall cell variant (TCV), characterized by worse outcome. Although generally cured by standard therapy that includes surgery, radioiodine therapy, and thyroid hormone replacement, PTC can progress to poorly differentiated or undifferentiated forms. PDTC and ATC are rare (5 and 2%, respectively) and are associated with poor prognosis, with survival reduced to a few months in the case of ATC [1]. Treatment of ATC represents a clinical challenge, as no effective therapies are presently available.

The inflammatory tumor microenvironment plays a critical role in different stages of development of many tumor types by affecting immune surveillance and responses to therapy. Cancer cells secrete several cytokines and chemokines, thus sustaining their growth and recruiting different leukocytes into the tumor site. In turn, pro-inflammatory cytokines produced by tumor-associated inflammatory cells can promote cancer progression [2]. Overall, the complex interplay between infiltrating immune cells and tumor cells has emerged as an essential feature of tumors. In this scenario, macrophages are key players: tumor-associated macrophages (TAMs) support cancer cell survival, proliferation, and invasion, and thus represent important new therapeutic targets [3–6].

The critical role of inflammation in thyroid tumors is well documented. Conditions predisposing to cancer (e.g. autoimmune Hashimoto’s thyroiditis), as well as causative genetic events (PTC-associated oncogenes), contribute to the construction of an inflammatory microenvironment. In in vitro models, thyroid oncogenes induce the production of inflammatory mediators including molecules such as CSF-1 and CCL2, which are able to recruit monocytes [7]. Macrophage infiltration is frequently detected in human thyroid tumors. The density of TAMs has been associated with larger tumor size and lymph node metastases in PTC [8, 9], and with tumor invasion and decreased survival in PDTC [10]. In ATC, TAMs constitute more than 50% of the tumor mass and form a very dense network in direct contact with intermingled cancer cells [11]. TAMs in thyroid tumors display an M2 phenotype (i.e. express CD163 and interleukin 10), and are likely to promote tumor progression and/or inhibit tumor elimination [12]. In line with this, pharmacological targeting of CSF-1/CSF-1R inhibits TAMs and impairs BRAF-induced thyroid cancer progression in BRAF-V600E transgenic mice [13]. Nevertheless, no functional in vitro studies dissecting the interplay between thyroid tumor cells and macrophages are available.

Oncogene-induced senescence (OIS) is a stable cell cycle arrest first identified in vitro, as response to the enforced expression of cancer-promoting genes in normal primary cells [14]. Later on, OIS was also detected in diverse precancerous tissues from humans and mice, and proposed to act as a barrier against malignant transformation in vivo [14–16]. Senescent cells display changes in morphology, increased β-galactosidase activity, formation of heterochromatin foci, up-regulation of p16^INK4a^, p21^CIP1^, and p53, and production of a complex secretome (known as senescence-associated secretory phenotype, SASP) that is characterized by a mixture of growth factors, inflammatory cytokines, and chemokines [17]. Under different conditions, SASP may restrain or promote cancer progression. On one hand, SASP counteracts cancer progression by inducing OIS in neighboring cells and by acting on the surrounding microenvironment, stimulating the innate and adaptive antitumor immune response (“senescence surveillance”), thus leading to tumor clearance [18–22]. On the other hand, SASP favors tumor progression by supporting proliferation of tumor cells and by creating an immunosuppressive microenvironment that facilitates tumor growth. Specifically, SASP increases myeloid-derived suppressor cells that are capable of inhibiting T-cell function [23]. Furthermore, there is some evidence that macrophages are key elements for execution of SASP-mediated effects [18].

We have previously demonstrated that OIS is involved in thyroid carcinogenesis. Through an in vitro approach we found that expression of PTC-associated oncogenes in primary human thyrocytes trigger senescence. Immunohistochemical analysis of thyroid tumors showed that OIS markers are up-regulated in early tumor stages, and progressively lost in more advanced and aggressive stages [24]. Therefore, we proposed senescence thyrocytes as a good in vitro model for the early stages of thyroid tumors [24, 25]. Furthermore, by using an inducible system of RAS-induced senescence in thyrocytes, we highlighted the link between senescence and inflammation [25]. Later on, other authors also proposed a role of OIS in PTC evolution by using in vitro and in vivo models of BRAFV600E-driven thyroid carcinogenesis [26, 27]. More recently, Kim et al. [28] demonstrated the involvement of senescent cells in the collective invasion and metastasis of PTC, thus highlighting their protumorigenic capacity. Despite the growing evidence of OIS involvement in thyroid cancer, little is known about the interaction of senescent thyrocytes with microenvironment; dissecting this issue is important to understand not only the reciprocal effects of thyroid cells and their microenvironment, but also the interplay between senescent and tumor cells mediated by components of the microenvironment. Herein, using an in vitro approach, we characterize the interplay between macrophages and senescent thyrocytes and tumor-derived cell lines, respectively modeling early and late thyroid tumor stages.

## Materials and methods

### Thyroid cell cultures and conditioned media preparation

ER:RAS human primary thyrocyte cultures have been previously described [25]. Nthy-ori 3–1 cell line (SV-40 immortalized normal human thyroid follicular cells) were cultured in RPMI 1640 medium (Gibco Life Technologies, Carlsbad, CA, USA). The PTC-derived K1 cell line was grown in DMEM: Ham’s F12: MCDB. The other cell lines used (PTC-derived BCPAP, TPC-1, NIM-1, and ATC-derived HTC/C3, FRO81–2, HOTCH, 8505-C, KAT-18) were maintained in DMEM (Gibco, Life Technologies, Carlsbad, CA, USA) medium. All cell lines were cultured in medium supplemented with 10% (v/v) heat-inactivated fetal bovine serum (FBS, EuroClone, Pero, Italy) as monolayers at 37 °C in a 5% CO_2_ humidified atmosphere. Cell lines were authenticated by short tandem repeat (STR) profiles using the StemElite ID System (Promega, Madison, Wisconsin, United States) by the Fragment Analysis Facility at Fondazione IRCCS Istituto Nazionale dei Tumori. Cells were routinely tested for mycoplasma (PCR Mycoplasma Detection Set, TAKARA Bio Inc., Kusatsu, Japan).

For the preparation of conditioned media, ER:RAS thyrocytes, untreated or treated with 4OHT, and thyroid tumor cells were cultured in their own medium until 80% confluence. The medium was discarded, cells were washed twice with PBS, and then incubated with 6/8 ml of fresh medium (complete for ER:RAS thyrocytes, supplemented with 5% FBS for tumor cell lines). After 24/48 h, the CM was collected, centrifuged, passed through 0.20 μm filters, and stored at − 20 °C until use.

### In vitro preparation of control and thyroid cell-polarized macrophages, and related conditioned media

Human monocytes were obtained from the leukocyte-rich component (buffy coat) of blood healthy donors using a protocol approved by the Ethical committee of the IRCCS Clinical and Research Institute Humanitas. Monocytes were purified through Ficoll and Percoll density gradients as previously described [29], and differentiated into macrophages by culture (10^6^/ml) in RPMI 1640 + 5% FBS, with 25 ng/ml hrM-CSF. At day 6, cells were stimulated with LPS (100 ng/ml) and IFN-γ (500 U/ml) to obtain M1-polarized macrophages, or with IL-4 (20 ng/ml) to obtain M2-polarized macrophages; unstimulated cultures were considered M0 macrophages. HrM-CSF, LPS, IFN-γ and IL-4 were from PeproTech, Rocky Hill, NJ, USA.

To obtain macrophages differentiated and conditioned by thyroid cell supernatants, monocytes (10^6^/ml) were cultured for 6 days in the presence of 30% of cell conditioned medium (CM) from either ER:RAS thyrocytes, treated with or without 4OHT, or different thyroid tumor cells, in the absence of exogenous hrM-CSF; at day 4, 30% of CM was replaced. At day 7, cultures were collected and centrifuged. Cell pellets were used for phenotypic analysis; supernatants (conditioned media) were passed through 0.20 μm filters and stored at − 20 °C until use.

### Wound healing assay

K1 cells were seeded in a culture insert (#80209, Ibidi, Planegg, Germany), placed in 24 well plates (6 × 10^4^ cells/chamber), and incubated overnight. Inserts were then removed to generate a 500 μm gap in the cell layer; following washing with PBS and supplementing with the indicated media, plates were re-incubated. To monitor gap closure, images were taken from at least 3 fields for each chamber at 3, 5, 8, and 24 h using Cell-IQ Imagen software (CM Technology Oy, Tampere, Finland). To quantify the closure, images were analyzed with the Scratch wound measurement tool of the Cell-IQ Analyser software (CM Technology Oy, Tampere, Finland); the % of closed area was calculated with the equation: (Start wound – wound [μm2])/Start wound × 100. Data and graphs were analyzed using GraphPad Prism 5.02.

### Enzyme-linked immunosorbent assay (ELISA)

Quantification of the levels of CCL17 and PGE2 in cell supernatants was measured by commercially available ELISA according to the manufacturer’s instructions: Human CCL17 Cat#: DY364 (R&D Systems, Space Import, Milan, Italy); Prostaglandin E2 ELISA Kit Cat#: 514010 (Cayman Chemicals, MI, USA).

### Flow cytometry

In vitro-differentiated macrophages were analyzed by flow cytometry on FACS Canto (BD Biosciences, Milan, Italy). Cells were pre-incubated with PBS 1% human serum to block FcRs for 30 min, washed and suspended in FACS buffer (PBS 0.5% BSA, 0.05% NaN3), and stained with the following mAbs: PerCP-Cy™5.5 Mouse Anti-Human HLA-DR, Clone G46–6; FITC Mouse Anti-Human CD206, Clone 19.2; BV421 Mouse Anti-Human CD163, Clone GHI/61. All mAbs were purchased from BD Biosciences (Milan, Italy).

### Gene expression analysis

Macrophage cultures and thyroid cell lines were analyzed with a customized TaqMan Low Density Array (Applied Biosystems, Foster City, CA, United States) with 91 inflammation-related genes [30]. PCR amplification was done in the micro-fluidic card sample block of an ABI Prism® 7900HT Fast Real-Time PCR System (Applied Biosystems, Foster City, CA, United States), following the manufacturer’s instructions. Data were calculated as the relative mRNA amount of each target gene over the 18S housekeeping gene and expressed as ΔCt, where ΔCt = Ct_gene_ – Ct_18S_. The fold-change of each mRNA target gene to the corresponding control population was calculated as 2^(−ΔΔCt)^, where ΔΔCt = ΔCt_target gene_ - ΔCt_relative control gene_. The threshold cycle Ct was automatically given by the SDS2.2 software package (Applied Biosystems, Foster City, CA, United States).

The expression of the PTGS2, CD163, and CD206 genes in human thyroid tissues was investigated in four public datasets available on NCBI’s Genome Expression Omnibus (GEO): GSE3467 [31], GSE6004 [32], GSE33630 [33, 34], and GSE76039 [35]. Raw intensity expression values obtained on Affymetrix U133 plus 2.0 array were processed and normalized as previously described [36] to generate a single series of 178 samples including 31 ATCs, 72 PTCs, 17 PDTCs, and 58 matched normal thyroids. Patients were stratified for PTGS2 and CD163 expression according to each gene median expression (high ≥ median; low < median). Gene expression heatmaps were visualized by the Clustergrammer tool [37]. Statistical analyses were performed using GraphPad Prism 5.02 software. Gene correlation was evaluated by Spearman’s correlation coefficient.

### Treatment with COX-2 inhibitors

To determine the optimal concentration of celecoxib (Sigma Aldrich, St Louis, MO, USA), ER:RAS thyrocytes and tumor cell lines were treated with increasing doses (0, 5, 10, 20, 30, 40, 60, 80 or 100 μM) for 24, 48, or 72 h. For each cell line the minimum effective and the maximum non-toxic dose was defined. Cell viability was measured using the Cristal violet staining assay [25].

For NS398 (Sigma-Aldrich, St. Louis, MO, USA), optimal concentrations for treatment were deduced from Park et al. [38]; cells were treated with 5 and 10 μM NS398 for 72 h.

### Statistical analysis

All statistical analyses were performed using GraphPad Prism software (version 5.02). Groups were compared using the two-tailed unpaired Student’s *t*-test or non parametric Mann-Whitney test. *P*-values < 0.05 were considered significant.

## Results

### In vitro model of senescent thyrocytes

As an in vitro model of senescent thyrocytes, we used an inducible system previously established and characterized in our laboratory based on primary human thyrocytes infected with a retroviral vector carrying an activated form of H-RAS^G12V^ fused to the 4-hydroxytamoxifen (4OHT)-responsive estrogen receptor ligand binding domain. Briefly, human primary thyrocytes were transduced with an *ER:RAS* retroviral vector; after 7 days of 4OHT treatment ER:RAS thyrocytes undergo senescence, as documented by the presence of senescence-specific markers, including growth arrest, morphology changes, and induction of SASP program. On the contrary, no changes occur in untreated cells, which continued proliferating [25]. Further characterization of our model is reported in Additional file 1: Supplementary results, and Additional file 2: Figure S1.

### Senescent thyrocytes and thyroid tumor cell lines trigger macrophage differentiation and M2-like polarization

**Fig. 1:**
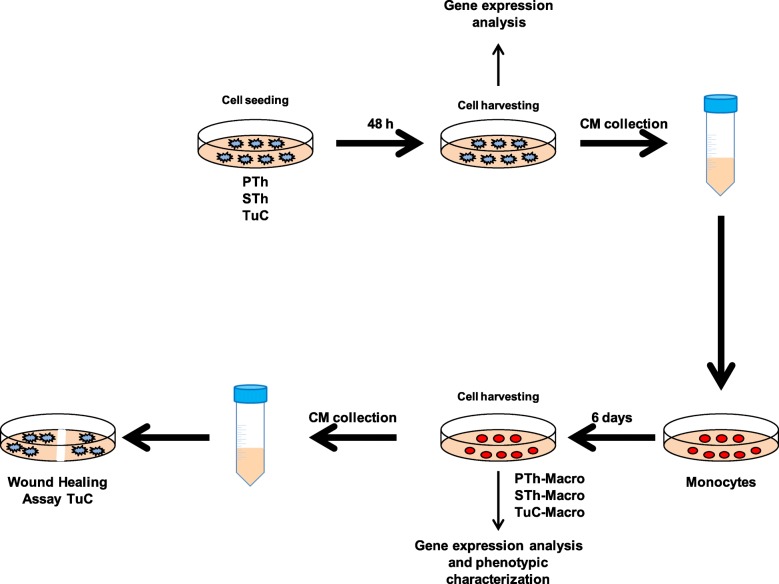
Flowchart of the experimental design. PTh: proliferating thyrocytes; STh: senescent thyrocytes; TuC: tumor cell lines; CM: conditioned medium; Macro: macrophages

The experiments hereafter described were performed according to the flowchart reported in Fig. 1. Similarly to other senescent cellular models, thyrocytes undergoing oncogene-induced senescence activate a SASP-like response [25]. To study the impact of the proteins secreted by senescent thyrocytes on cells of innate immunity, conditioned medium (CM) from ER:RAS thyrocytes untreated (proliferating thyrocytes, PTh) or treated with 4OHT for different times (senescent thyrocytes, STh) was used on human monocytes, and the effect on cell differentiation and functional polarization was investigated.

Human purified monocytes from healthy donors were exposed to CM (30% v/v) of senescent or proliferating thyrocytes (SThCM, PThCM) in the absence of exogenous growth factors for 6–7 days. Interestingly, both types of CM induced full macrophage differentiation similar to that of control M0 macrophages (obtained by exposing monocytes to hrM-CSF; mean 58+/− 5% CD68+ macrophages, relative to the starting population, as determined in more than 10 experiments). In line with this, both PTh and STh express substantial levels of CSF-1 (Additional file 3: Figure S2a).

**Fig. 2:**
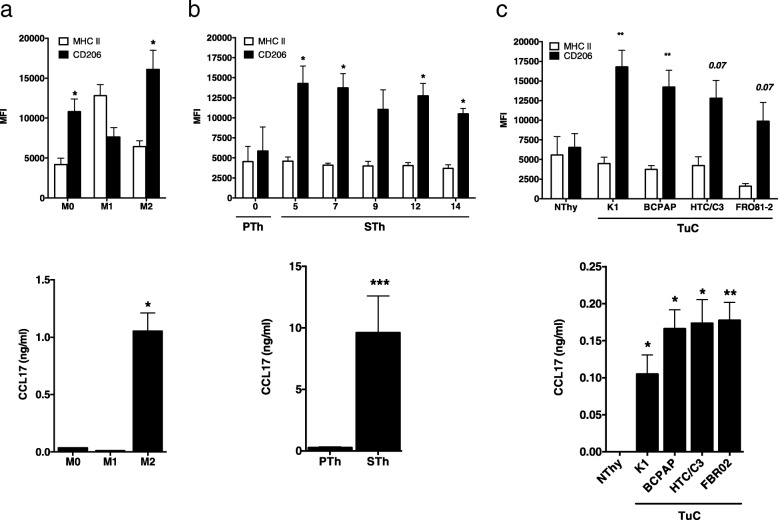
Conditioned medium of senescent thyrocytes and thyroid tumor cell lines triggers M2-like macrophage polarization. In vitro hrM-CSF-generated control macrophages (**a**), human purified monocytes treated with the conditioned medium of proliferating (PTh) and senescent (STh) thyrocytes, collected at the indicated time points after 4OHT treatment (**b**), and normal Nthy-ori 3–1 (Nthy) and tumor (TuC) thyroid cell lines (**c**) were analyzed by FACS for the expression of MHCII and CD206 (top panels) and by ELISA for the secretion of CCL17 (bottom panels). Histograms represent mean + standard deviation of 3–4 independent experiments. Statistical significance was determined by unpaired t test *p < 0.05, ** *p* < 0.01, ***p < 0.001. *MFI*: mean fluorescence intensity

Macrophages were subjected to phenotype analysis by flow cytometry; as shown in Fig. 2a, top panel, as expected, control M1 macrophages (obtained by stimulation with LPS and IFN-γ) expressed higher levels of MHC II molecules compared to non-polarized cells (M0 macrophages), while control M2 macrophages (obtained by stimulation with IL-4) expressed higher levels of the mannose receptor (CD206). Macrophages differentiated by exposure to SThCM displayed a M2-like phenotype, expressing low MHC II molecules and higher CD206 than cells exposed to PThCM (Fig. 2b, top panel).

A similar approach was used to evaluate the effect of CM from 10 different thyroid cell lines on monocytes, in the absence of exogenous growth factors. CM from immortalized normal thyrocytes (Nthy), and 9 thyroid carcinoma cell lines (TuC; PTC-derived: K1, BCPAP, TPC1 and NIM-1; ATC-derived: HTC/C3, FRO81–2, HOTCH, 8505-C, and KAT-18) were analyzed as described above. The tumor cell lines K1, BCPAP, HTC/C3, and FR081–2, and the immortalized cell line Nthy induced differentiation of macrophages and expressed CSF-1 (Additional file 3: Figure S2b). Phenotype analysis showed that macrophages differentiated in the presence of CM derived from the four thyroid tumor cell lines (Tumor cells-Conditioned macrophages, TuC-Macro), expressed low MHC II molecules and higher CD206 compared with macrophages exposed to Nthy-derived CM (Fig. 2c, top panel); this indicates that they display an M2-like phenotype.

The M2-like polarization of conditioned macrophages was further confirmed by ELISA assays for the secretion of CCL17, a chemokine produced by M2 macrophages (Fig. 2a, bottom panel), typically recruiting Th2 cells. M2-like STh-Macro and TuC-Macro produced higher CCL17 levels than macrophages differentiated, but not polarized, after exposure to CM from proliferating (PTh) or immortalized (Nthy) thyrocytes, respectively (Fig. 2b and c, bottom panels) .

**Fig. 3:**
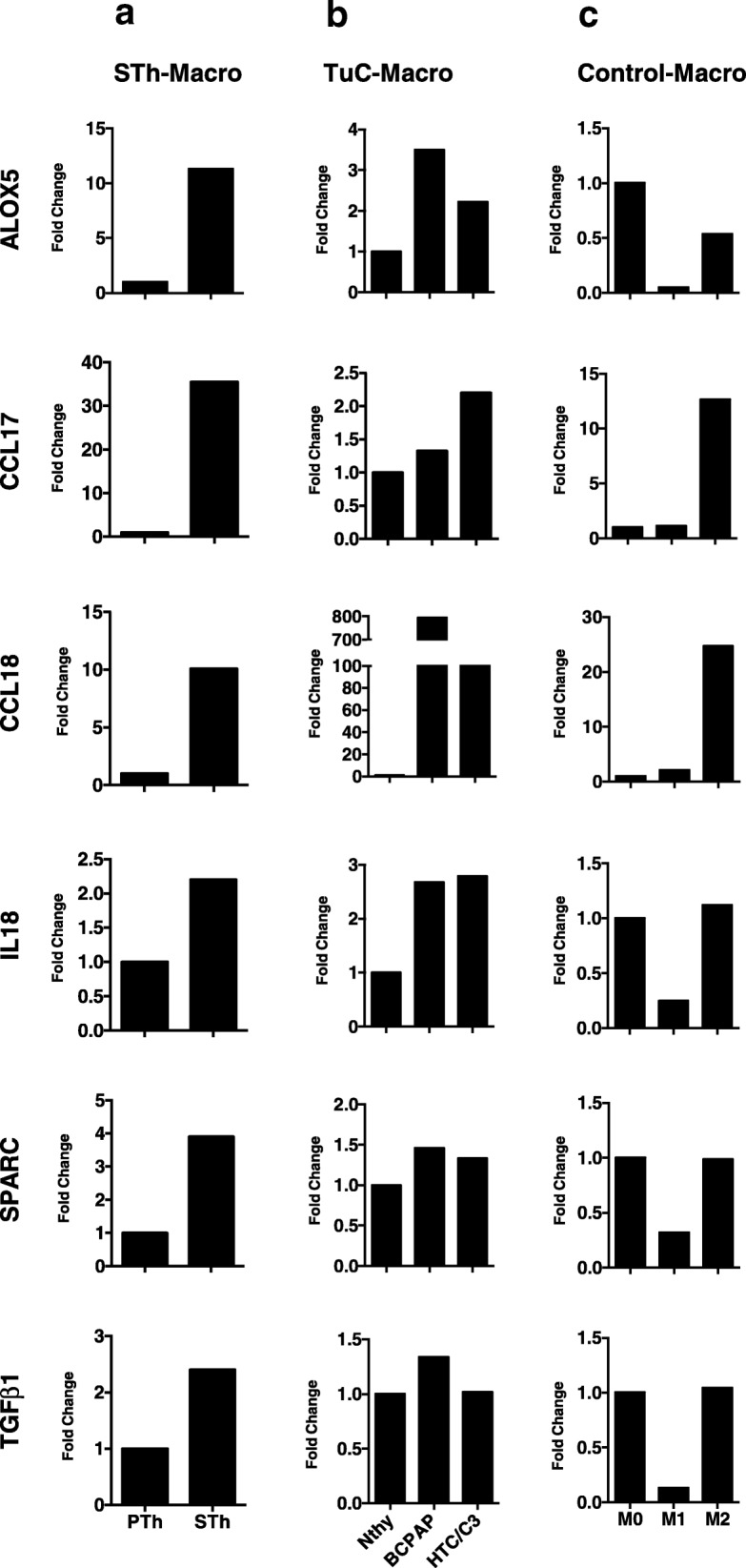
Monocytes exposed to senescent thyrocytes and thyroid tumor cell lines express M2-related genes. qRT-PCR analysis of genes related to M2 macrophage phenotype in monocytes treated with the conditioned medium from: **a** proliferating (PTh) and senescent (STh) thyrocytes; **b** normal thyroid cells (NThy) or tumor thyroid cell lines: BCPAP and HTC/C3. **c** Control macrophages: M0 (hrM-CSF-generated), M1 (stimulated with LPS and IFN-γ) and M2 (stimulated with IL-4). Results were normalized with 18S RNA levels and expressed as fold change relative to their respective controls: PTh in (**a**); NThy in (**b**), M0 in (**c**)

To have a broader view of the effects of the CM derived from both senescent thyrocytes and tumor cell lines on macrophage polarization, we performed gene expression analysis using a customized TaqMan Low Density Array containing 91 genes related to immune-inflammatory pathways. ALOX5, CCL17, CCL18, IL18, the matrix-related molecules osteonectin (SPARC) and TGF-β1 were among the genes upregulated in STh-Macro relative to PTh-Macro (Fig. 3a); these genes are typically more expressed in control human M2-polarized than in M1-polarized macrophages (obtained by stimulation in vitro with IL-4 or LPS and IFN-γ, respectively) (Fig. 3c). Similarly, TuC-Macro, relative to macrophages exposed to Nthy CM, also showed upregulation of these genes, most prominently of ALOX5, CCL18, and IL18 (Fig. 3b). These results corroborated the M2-like phenotype of Sth- and TuC-Macro.

**Fig. 4:**
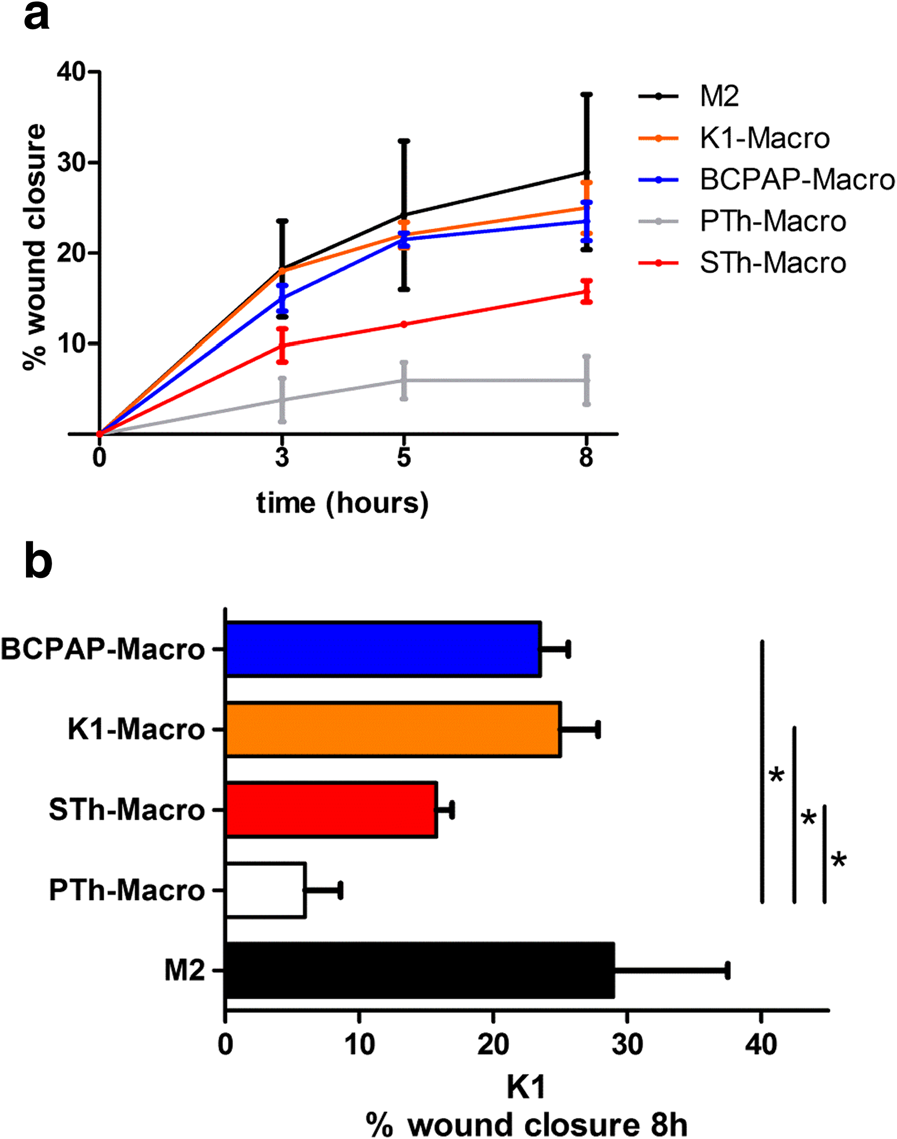
Conditioned medium of M2-like macrophages enhances the migration property of K1 cells. Wound healing assay was performed on K1 cells treated with conditioned medium (CM) from macrophages exposed to CM from BCPAP (BCPAP-Macro), K1 (K1-Macro), senescent (STh-Macro) and proliferating thyrocytes (PTh-Macro), or from control M2 macrophages. The graph shows the percentage of wound closure quantified at the indicated time points (**a**) and specifically 8 h post-wound (**b**). Error bars represent standard deviation of two independent experiments. Statistical significance was determined by unpaired t test. *p < 0.05

It has been reported that TAMs from thyroid carcinoma increase the in vitro migration ability of thyroid tumor cell lines, whereas no effect was detected on proliferation/apoptosis [39, 40]. We investigated the effect of the above described M2-like macrophages on the migration capability of K1 cell line by a wound healing assay. We first tested the effect of CM derived from in vitro-differentiated control M0, M1, and M2 macrophages and found that CM from M2 macrophages increased the migration of K1 cells more than CM from M0 or M1 macrophages (Additional file 4: Figure S3). Next, we similarly analyzed the effect of CM from TuC-Macro (obtained through K1 and BCPAP) and from STh- or PTh-Macro on K1 cells. The migratory effect of M2-like STh- and TuC-Macro was significantly higher than PTh-macro (differentiated but not polarized), and comparable to that of control M2-polarized macrophages (Fig. 4).

### Identification of factors involved in M2-like polarization induced by senescent thyrocytes and tumor cells

To gain insight into possible secreted factors mediating the M2-like polarizing activity of different thyroid cell-derived CM, we performed gene expression analysis of thyroid tumor cell lines, Nthy, senescent and proliferating thyrocytes with the TaqMan Low Density Array, focusing on genes upregulated in at least one cell model (Table 1).

**Table 1:** Expression levels of genes related to immune-inflammatory pathways in thyroid cells

	PTh	STh	Nthy	K1	BCPAP	HTC/C3
ALOX5	40,1	15,2	9,3	ND	6,5	42,3
CCL20	70,9	368,3	895,7	202,3	406,4	19,6
CCL26	14,2	9,6	12,7	5,5	17,1	400,7
CCL3	0,2	3,1	1,6	50,1	ND	186,6
CCL4	0,2	2,8	3,0	1,1	ND	38,8
CSF-1	1515,8	1521,2	1771,5	695,7	3515,0	2501,5
CXCL1	8124,4	6413,7	3057,2	10721,2	63634,1	95057,1
CXCL10	6,4	9,2	3,8	ND	405,2	10,0
IL10	ND	ND	ND	ND	ND	ND
IL12A	53,7	55,3	24,8	1,8	209,7	26,4
IL18	697,1	68,8	121,9	412,0	2204,5	10418,2
IL1B	3,1	24,5	2,5	19684,9	8636,1	1699,8
IL23A	2,2	3,1	8,0	1,6	72,4	220,1
IL24	4,6	2,4	25,6	478,7	262,7	57,6
IL6	478,0	56,8	1050,0	431,4	4878,7	14460,2
IL8	23515,8	33391,8	11413,4	3675,3	70817,6	41986,9
PTGS2	8,1	22,5	1,7	58,5	1665,5	325,2
SIGIRR	16,6	19,7	82,8	29,7	207,8	174,7
SPP1	112,8	362,0	12,1	39,4	113,0	694,0
TGFB1	5190,3	5722,8	2148,8	1956,2	15559,7	1006,1
TNF	2,0	7,6	131,4	90,6	673,5	1629,2
VEGFA	8567,8	21958,5	1406,6	7613,1	5161,8	2964,5

qRT-PCR analysis of genes related to immune-inflammatory pathways in: proliferating (PTh) and senescent (STh) thyrocytes; normal thyroid cells (NThy) and tumor thyroid cell lines (K1, BCPAP and HTC/C3). Results are expressed as fold-change relative to 18S housekeeping gene

Of note, no genes widely recognized to induce M2 polarization were found: IL10 was undetectable in all samples; TGFB1, IL6, and VEGFA were also expressed by proliferating thyrocytes and Nthy cells, which do not induce M2-like polarization (see previous paragraph).

**Fig. 5:**
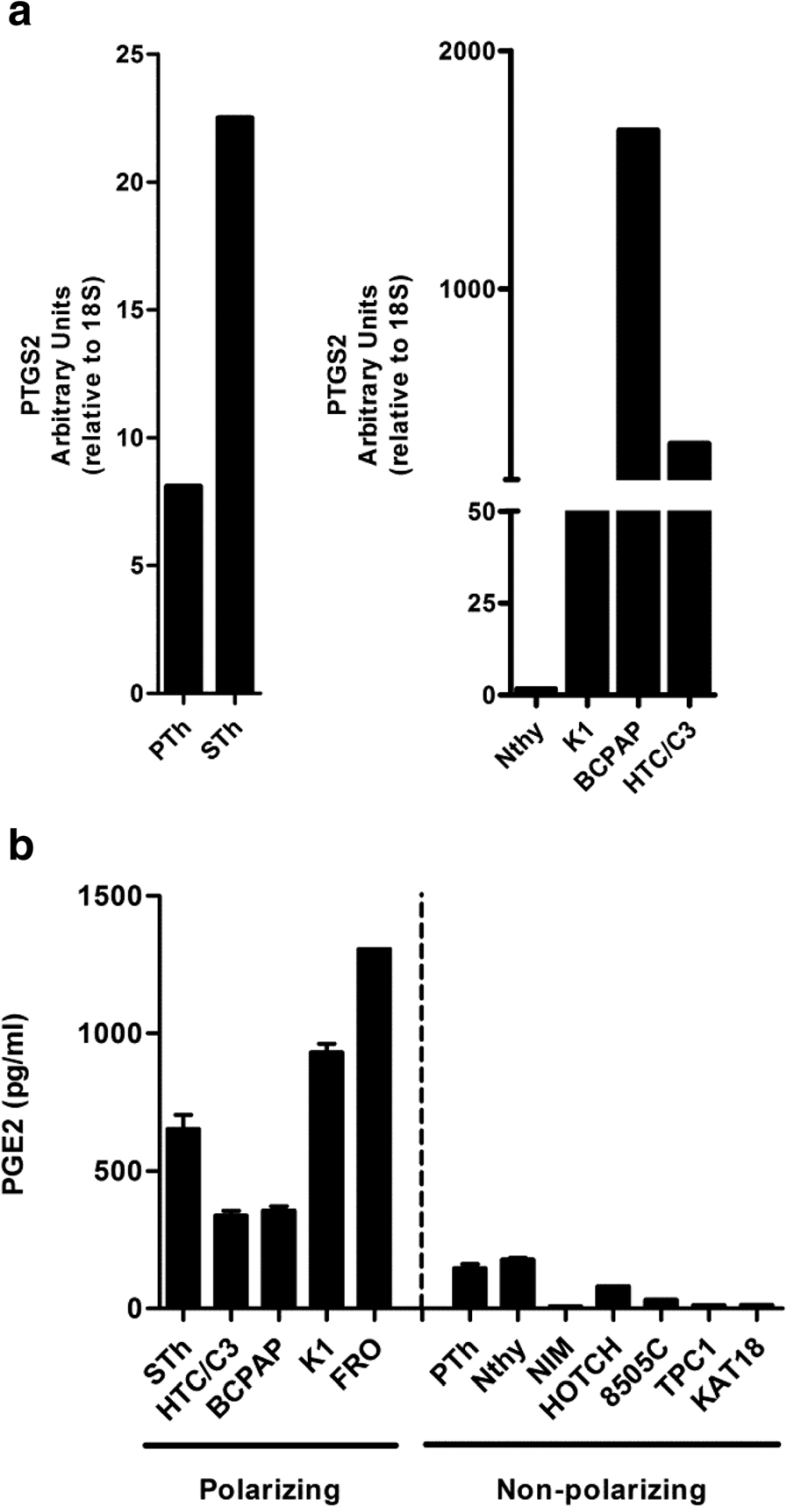
Thyroid cells with M2-like polarizing activity express the PTGS2 gene and secrete PGE2. **a** qRT-PCR analysis of PTGS2 in senescent (STh) and proliferating thyrocytes (PTh), and in M2-polarizing thyroid cell lines (K1, BCPAP, HTC/C3). **b** PGE2 levels, determined by ELISA, in the conditioned medium from senescent (STh) and proliferating thyrocytes (PTh), and from polarizing and non-polarizing thyroid cell lines

The genes that were overexpressed in both senescent thyrocytes and at least one tumor cell line included genes coding for: the chemokines CCL3 and CCL-4, the cytokine IL-1β, the matrix-related protein SPP1, and the enzyme PTGS2. Among these, we focused on PTGS2 as it was overexpressed in all polarizing thyroid cells (Fig. 5a).

PTGS2 gene encodes the inducible form of the prostaglandin-endoperoxide synthase enzyme, or cyclooxygenase-2 (COX-2), involved in the production of prostaglandin E2 (PGE2). As prostaglandins are known to have immune-suppressive effects on some types of immune cells [41], we hypothesized that they might be involved in the M2-like polarization of STh- and TuC- Macro. We used ELISA to analyze the production of PGE2 in thyroid cell CM. PGE2 secretion was high only in tumor thyroid cell lines with M2-like polarizing ability and in senescent thyrocytes; on the contrary, the non-polarizing tumor cell lines, as well as PTh and Nthy, produced low levels of PGE2 (Fig. 5b).

**Fig. 6:**
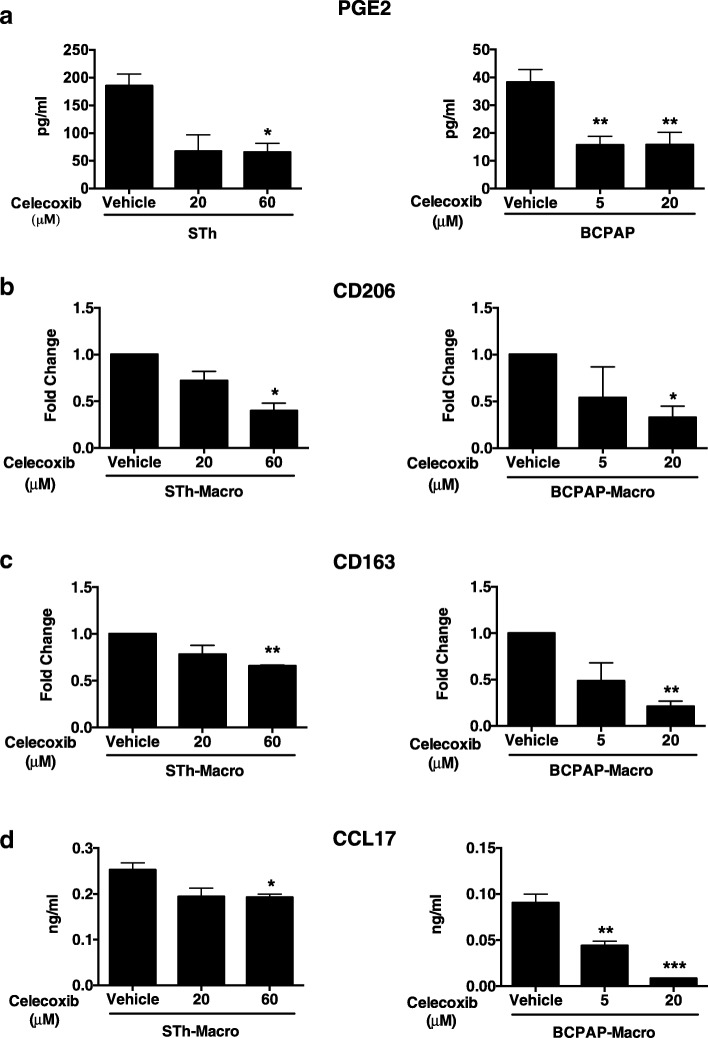
Celecoxib inhibits PGE2 production and M2-like polarizing ability of senescent thyrocytes and BCPAP thyroid cells. PGE2 secretion, determined by ELISA, of senescent thyrocytes (STh) and BCPAP cells treated with celecoxib at the indicated doses (**a**). Macrophages induced by CM from samples in (**a**) were analyzed for CD206 and CD163 expression by FACS (**b**, **c**) and for the secretion of CCL17 by ELISA (**d**). Statistical significance was determined by unpaired t test. *p < 0.05, **p < 0.01, ***p < 0.001

To verify if PGE2 was the factor responsible for the M2-like polarizing ability, we treated senescent thyrocytes and the BCPAP cell line with celecoxib, a specific inhibitor of COX-2 activity. Secretion of PGE2 was efficiently reduced after celecoxib treatment (Fig. 6a). To investigate if inhibition of PGE2 production impacts the M2-like polarizing ability, the CM of STh and BCPAP cells, untreated or treated with celecoxib, was used to treat human purified monocytes from healthy donors in the absence of exogenous growth factors. FACS analysis of differentiated macrophages showed that the expression of the two M2-specific surface markers, CD206 and CD163 was significantly reduced in macrophages exposed to CM of STh and BCPAP treated with celecoxib, suggesting that PGE2 has a role in the M2-polarization activity of both cell models (Fig. 6b and c). In line with these findings, also the secretion of the chemokine CCL17 was significantly reduced (Fig. 6d).

To confirm the role of the secreted factor PGE2 in M2-polarizing ability, we used NS398, another specific inhibitor of COX-2 activity. NS398 treatment efficiently reduced PGE2 production in BCPAP and K1 cells (Additional file 5: Figure S4a). CM of the cell lines, untreated or treated with NS398, was used to treat human purified monocytes from healthy donors, in the absence of exogenous growth factors. Analysis of CD206 expression and CCL17 secretion indicated that the polarizing activity of CM from NS398–treated cells was reduced compared to controls (Additional file 5: Figure S4b).

Overall, these results demonstrate that the M2-polarizing ability of senescent thyrocytes and thyroid tumor cells is dependent on secretion of PGE2.

### PTGS2 and M2 markers are co-expressed in a significant fraction of human thyroid carcinoma

To test the co-occurrence of PTGS2-expressing thyroid tumor cells and M2-macrophages in human specimens, we assessed the expression levels of PTGS2 and the M2 marker CD163 in the trascriptomes of a series of 178 human thyroid tissues, publicly available on GEO, and including 58 normal thyroids, 72 PTCs, 17 PDTCs and 31 ATCs.

**Fig. 7:**
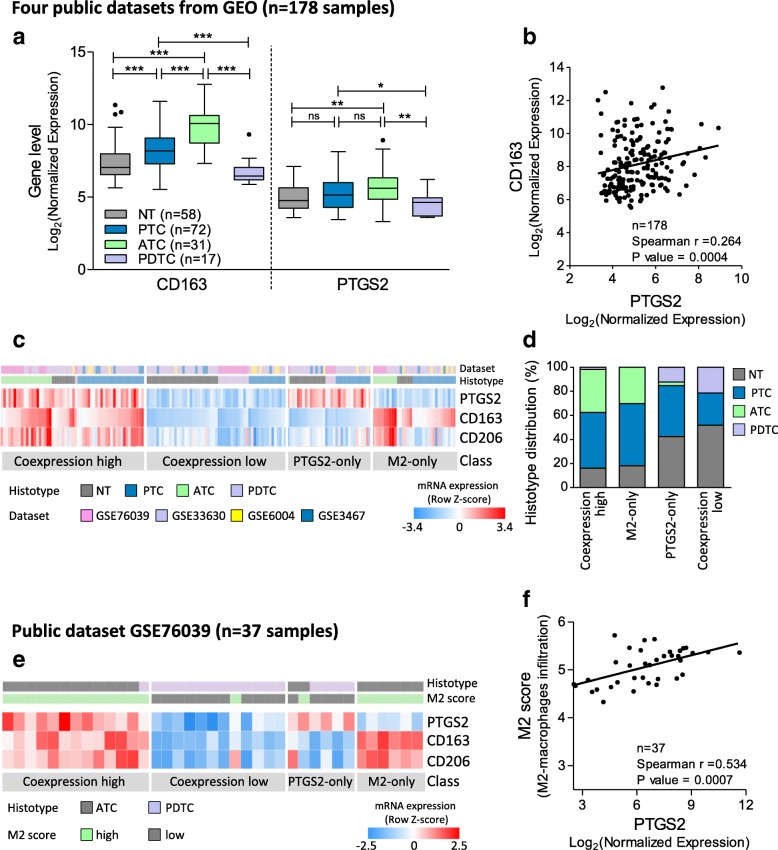
PTGS2 and M2 markers expression in human thyroid tissues. **a** Boxplots showing PTGS2 and CD163 gene expression in normal thyroids and tumor samples derived from GEO datasets (GSE3467, GSE6004, GSE33630 and GSE76039). Statistical significance by Mann Whitney test; * *P* < 0.05, ** *P* < 0.005, *** *P* < 0.0001. **b** Correlation by Spearman coefficient between PTGS2 and CD163 expression in the same series. **c** Heatmap showing PTGS2 and M2 markers (CD163 and CD206) expression across the same series; mRNA levels were expressed as normalized data (row Z-score). Class was established based on PTGS2 and CD163 median expression (high ≥ median; low < median); class coexpression high: coordinated PTGS2 and CD163 high; class coexpression low: coordinated PTGS2 and CD163 low; class PTGS2-only: PTGS2high and CD163 low; and class M2-only: CD163 high and PTGS2 low. **d** Barplot showing histotype distribution across PTGS2/CD163 expression classes. **e** Heatmap showing PTGS2 and M2 markers (CD163 and CD206) expression in GSE76039 dataset; PTGS2/CD163 expression class, histotype and M2 score (indicative of macrophages infiltration) is indicate for each sample. **f** Correlation between PTGS2 expression an M2 score in GSE76039 dataset

PTCs and ATCs exhibited significant and higher expression of CD163 compared with normal thyroid (Fig. 7a), in agreement with previous reports [8–11], and a trend toward increased expression of PTGS2, even though less striking. Of note, the PDTC samples included in this set displayed low expression of both PTGS2 and CD163. In the same cohort, we found weak but significant positive correlation between PTGS2 and CD163 (Fig. 7b). When we explored the coordinated expression of these two genes, we could stratify patients in 4 classes (Fig. 7c): two classes of co-expression (high or low) where PTGS2 and CD163 displayed a strong correlation (Spearman *r* = 0,754; *P* < 0.0001), and two classes where just one of the two genes was highly expressed. In addition, CD206, another M2 marker, highly correlated with CD163 across all classes (Spearman *r* = 0,695; *P* < 0.0001) (Fig. 7c). Interestingly, we found that a significant number of samples (112/178, 63%) displayed co-expression of PTGS2 and M2; among these, PTCs and ATCs were the prevalent tissue types (82%, Fig. 7d), harboring high levels of both genes (class co-expression high). While PTCs were heterogeneously distributed in all classes, most ATCs (30/31, 96%) fell into the classes with high CD163 levels (i.e. class co-expression high and M2-only, Fig. 7d), according to the reported macrophage infiltration [11]. A similar classification and significant correlation between PTGS2 and CD163 was also observed in the large cohort of TCGA [42] comprising 486 PTCs (Additional file 6: Figure S5).

Further data supporting a link between PTGS2 and macrophages were found in one of the four datasets investigated (GSE76039); in this series, the authors established an M2 score indicative of macrophage infiltration based on a gene signature overexpressed in M2 macrophages, and reported a high M2 score in ATCs [35]. When we combined this M2 score with expression data, we found not only concordance between the M2 score and CD163/CD206 expression (Fig. 7e), but also a significant correlation between M2 score and PTGS2 expression (Fig. 7f).

Taken together, these data show co-expression of PTGS2 and M2 markers in a significant fraction of human thyroid cancer tissues, supporting our in vitro findings.

## Discussion

**Fig. 8:**
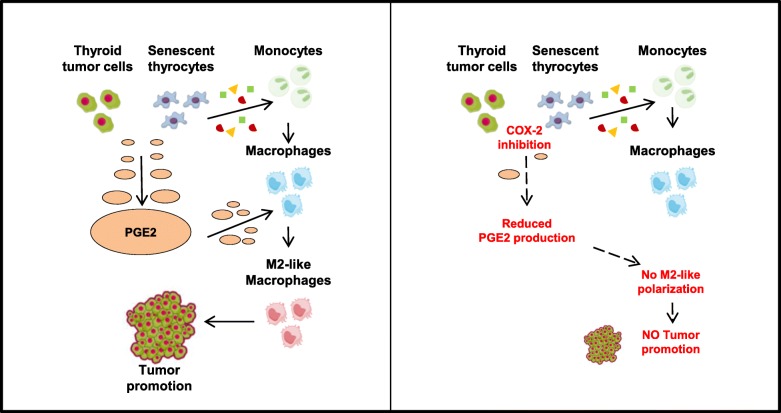
Graphical summary. The factors secreted by thyroid tumor cells and senescent thyrocytes promote the differentiation of monocytes into macrophages. Among the soluble proteins released, PGE2 is able to promote the switch of differentiated macrophages to a M2-like phenotype, which contribute to thyroid tumor progression. The pharmacological inhibition of the PGE2 producing enzyme COX- 2 inhibits PGE2 secretion and impairs the M2-like polarizing ability of thyroid cells

In this work, we investigated the effects of senescent and tumor thyroid cells on innate immunity cells, in particular monocytes, and dissected the mechanisms involved. Freshly isolated human monocytes were exposed to cell-conditioned media from senescent primary thyrocytes or established tumor cell lines, modeling early and late thyroid tumor stages, respectively. Our results demonstrate that CM from both senescent and tumor cells induce macrophage differentiation and clear cut M2-like protumoral polarization, and that this was mediated by PGE2 (Fig. 8). Analysis of publicly available datasets showed co-expression of PTGS2 and M2 markers in a consistent fraction of human thyroid cancer tissues.

We found that proliferating and senescent thyrocytes, the immortalized cell line Nthy, and four of nine thyroid tumor cell lines induced macrophage maturation; all of them produced CSF-1, the major growth and differentiation factor for monocytes. This finding is not surprising, because, due to the fundamental trophic role of macrophages, several tissues, including thyroid, have evolved to produce factors (such as CSF-1) that recruit and activate them, in order to maintain homeostasis.

Phenotypic and functional differences were, however, noted among the macrophage populations obtained. Cells exposed to proliferating thyrocytes or Nthy CM had an M0-like phenotype, while cells exposed to senescent thyrocytes or tumor cells CM (STh- and TuC-Macro) showed M2-like polarization (low MHC II, high CD206 or CD163 and high CCL17 production) and displayed tumor promoting activity.

TAMs have been frequently detected in PTC [8, 10], PDTC [10], and ATC, where they constitute greater than 50% of the tumor mass and are connected in a network throughout tumor tissue [11]. TAMs in thyroid cancers display an M2-like phenotype and are likely to promote tumor progression and/or inhibit tumor elimination [8, 11–13]. However, at variance with the mechanisms underlying macrophage recruitment to the tumor site [13], those regarding macrophage education to M2-like phenotype by thyroid tumor cells have been poorly investigated. Nevertheless, this represents an important issue, which could unveil possible tumor microenvironment targets, particularly relevant for ATC.

Our data demonstrate that both senescent cells and tumor cell lines induce M2-like polarization. Whereas the capacity of tumor cell lines is expected, based on the above reported in vivo studies, the ability of senescent thyrocytes to trigger M2-like polarization is novel, and suggests the capacity of senescent cells to promote a protumoral environment.

Through SASP, senescent cells act in opposing ways to restrain or to promote carcinogenesis. SASP factors released by senescent cells are able to act on the surrounding microenvironment triggering an immune-mediated antitumor response [18]. In contrast, SASP can also promote the growth of cancer cells, as well as tumor evasion of immune surveillance [43]. The relationship between senescent cells and the immune system is complex and incompletely understood, and the factors that control the pro- and anti-tumor surveillance activities remain to be identified.

Senescent cells are present within thyroid tumors, and their number inversely correlates with progression. Although more abundant in early stage PTMC, senescent cells are also present in malignant tumors [24, 28]. Recently, Kim et al. proposed a role for senescent cells in invasion and metastasis of PTC, and identified the SASP components involved; nevertheless, they did not investigate the possible contribution of the microenvironment in execution of the SASP effects [28]. Our data suggest that the protumoral effect of senescent thyrocytes can be mediated by macrophages. Indeed, we have shown that M2-like STh-Macro, similarly to M2-like TuC-Macro, increase the migration ability of the K1 thyroid tumor cell line. Of note, similar effects were observed by exposing thyroid tumor cell lines to TAMs isolated from PTC tissues [39].

To identify the mediators of M2-like polarization by thyroid cells, we performed gene expression analysis. We excluded the well-known immunosuppressive cytokines IL-10 and TGF-β1; the former because it is not produced by polarizing cells, and the latter because it is also produced by non-polarizing cells. We found high expression of PTGS2 in all M2-like-polarizing cells compared with non-polarizing thyroid cells; this strongly indicates that PGE2, the final product of COX-2 enzymatic reaction, may be involved. Our hypothesis was confirmed by the use of the COX-2 inhibitors (celecoxib and NS398), which reduced the ability of thyroid cells to induce M2-like-polarized macrophages.

COX-2 converts arachidonic acid to prostaglandins. COX-2 overexpression has been reported in most types of cancer, where it acts to promote cancer cell survival, growth, and angiogenesis. Targeting the COX-2/PGE2 pathway could provide a promising approach for cancer therapy, especially when it is used as an adjuvant with appropriate chemotherapeutic agents [44]. In accordance with this, the antitumor effect of celecoxib has been demonstrated both in vivo and in vitro [45, 46].

Prostaglandins have been proposed to play a critical role in the interplay between tumor cells and immune cells in the tumor microenvironment. PGE2 is known to have suppressive effects on leukocytes, especially antigen-presenting cells, and to favor the development of MDSC, as well as the induction of Treg cells [47–50]. High expression of PGE2 in TAM-rich tumors has been reported, but the exact role of PGE2 on the functional polarization of differentiating or mature macrophages has been investigated in only a few studies, and none related to thyroid cancer.

Several studies indicate that PGE2 plays an important role in the initiation and maintenance of M2 polarized TAMs, as well in promoting the suppression of M1 polarity. Nakanishi et al. reported that PGE2 initiates and maintains the M2-like polarization of macrophages infiltrating intestinal tumors in the genetic APC−/+ model [51]. In breast cancer, using in vitro and in vivo studies, it has been demonstrated that the feed-forward loop between COX-2 and PGE2 is essential for the modulation of TAMs towards immune suppressive and anti-inflammatory effectors [52, 53]. Thus, solid evidence demonstrates that the COX-2/PGE2 circuit in cancer cells is an important mechanism of tumor immuno-evasion [38, 41, 54].

Overall, our data are in line with previous literature results and extend the observation of PGE2-mediated immune suppression in thyroid cancer. More interestingly, we show in this study that the secretome of senescent thyrocytes also has a similar effect, and therefore the local availability of PGE2 may affect macrophages at both early and late stages of thyroid carcinogenesis. Of note, our finding that PGE2 is secreted by senescent thyrocytes is in agreement with previous studies in which PGE2 was identified as a soluble factor associated with SASP in other in vitro senescence models [21].

Several lines of evidence support a role of COX-2/PGE2 pathway in thyroid carcinogenesis. We have previously shown that the expression of PTC-associated oncogenes in primary [55] or immortalized human thyrocytes (our unpublished results) activates a transcriptomic inflammatory program, including COX-2. In vitro studies with the COX-2 inhibitor NS398 suggest a role for this enzyme in the proliferative and invasive ability of PTC cells [56, 57]. Scarpino et al. reported overexpression of COX-2 in PTC. Of note, COX-2 expression was significantly higher at the invasion front, highlighting its contribution to the invasive capacity of PTC cells [58]. Sun et al. reported that PTC produce PGE2, and overexpress both COX-2 and the EP4 PGE2 receptor [59]. Moreover, COX-2 expression is associated with recurrence in patients with both PTC and FTC [60]. More recently, Park et al. suggested a protective function for PGE2 secreted by thyroid cancer cells, allowing them to evade immune surveillance by suppressing the cytolytic activity of NK cells in the tumor microenvironment [38].

In agreement with the reports mentioned above, by analyzing public datasets, we detected not only high levels of PTGS2 mRNA in PTC and ATC, but also co-expression of PTGS2 and macrophage markers in a consistent fraction of thyroid tumors (class co-expression high). This highlights the link between PTGS2-expressing thyroid tumor cells and M2-macrophages, further corroborated by tissues displaying low expression of both markers (class co-expression low). Along with tissues characterized by consistent expression of PTGS2/M2 markers (63%, class co-expression), we found two subgroups of samples displaying high expression of only one of the two elements, which could represent tissues where macrophage differentiation/polarization is mediated by factors other than PTGS2/PGE2 (class M2-only), or tissues with inflammatory conditions not directly related to macrophages (class PTGS2-only). Of note, we found that PDTC of the analyzed series display low infiltration by macrophages, in line with a very recent study reporting poor or absent immune infiltration, including macrophages, in PDTC [61].

Overall, our results strongly suggest a protumoral effect of PGE2 mediated by its action on macrophages. Thus, COX-2 inhibition might represent a therapeutic strategy which, by modulating the activity of TAMs, would restore an antitumor immune response and counteract thyroid tumor growth. This is particularly relevant for ATC, in which TAMs constitute more than 50% of tumor mass, and for which no effective therapeutic options are available.

## Conclusions

Our in vitro results, summarized in Fig. 8, demonstrate that both senescent thyrocytes and thyroid tumor cell lines trigger M2-like macrophage polarization, and that this event is related to the up-regulation of COX-2 and consequent production of PGE2. This suggests that the interaction with macrophages in the microenvironment may occur at both early and late thyroid tumor stages, and favor tumor progression.

The demonstration that thyroid senescent cells trigger a protumoral microenvironment corroborates the notion that their presence within a tumor mass is detrimental, and supports the hypothesis that targeting senescent cells and their effectors could represent a therapeutic strategy for thyroid tumors.

Our in vitro results are supported by the co-expression of PTGS2 gene and M2 markers detected in a significant fraction of human thyroid carcinoma, thus highlighting the possibility to counteract tumor growth through COX-2 inhibition. This is particularly relevant for ATC, which feature high TAM infiltration, and whose treatment represents an unmet clinical need.

## Additional files

Additional file 1: Supplementary Results and Methods. (DOCX 16 kb)

Additional file 2: **Figure S1.** Detection of senescence markers in ER:RAS human primary thyrocytes untreated or treated with 4OHT. Cells were analyzed by WB for the expression of ER:RAS and p16^INK4a^ proteins (β-actin represents loading control), and by BrdU incorporation assay for cell proliferation. Cells were treated with 4OHT for 4 days (a); for 24 or 48 h and monitored for the presence of senescence features at 7 or 14 days (b). In (c), the determination of the minimum 4OHT dose capable to induce thyrocyte senescence was assessed; in WB, values represent band densitometric analysis, normalized to β-actin loading control. For all BrdU experiments, bars represent mean + the standard deviation of five technical replicates RLU: relative luminescence unit (PDF 180 kb)

Additional file 3: **Figure S2.** qRT-PCR analysis of CSF-1 transcript levels normalized with the 18S RNA levels in: (a) proliferating (PTh) and senescent (STh) thyrocytes; (b) normal thyroid cell (Nthy) and tumor thyroid cell lines (K1, BCPAP, HTC/C3 and FRO81–2). (PDF 60 kb)

Additional file 4: **Figure S3.** Wound healing assay performed on K1 cells treated with conditioned media from M0, M1 and M2 control macrophages, or with media containing 0% or 10% FCS. The graph shows the percentage of wound closure quantified at the indicated time points (a) and specifically 8 h post-wound (b). Error bars represent standard deviation of four independent experiments. Statistical significance was determined by unpaired t test. ** *p* < 0.01, ***p < 0.001. (PDF 126 kb)

Additional file 5: **Figure S4.** NS398 inhibits PGE2 production and M2-like polarizing ability of BCPAP and K1 thyroid cells. (a) PGE2 secretion, determined by ELISA, of BCPAP and K1 cells treated with NS398 at the indicated doses. (b) Macrophages induced by CM from samples in (a) were analyzed for CD206 expression by FACS and for the secretion of CCL17 by ELISA. Statistical significance was determined by unpaired t test. *p < 0.05, **p < 0.01, ***p < 0.001. (PDF 10 kb)

Additional file 6: **Figure S5.** PTGS2 and M2 markers expression in PTCs from TCGA dataset. (a) Heatmap showing PTGS2 and M2 markers (CD163 and CD206) expression across 486 PTCs from TCGA study. Normalized RNA sequencing data of the three genes were downloaded from cBioPortal for Cancer Genomics (www.cbioportal.org; accessed January 2019). Class was established based on PTGS2 and CD163 median expression as described in Fig. 7. (b) Correlation by Spearman coefficient between PTGS2 and CD163 expression in the same cohort. (PDF 208 kb)

## Abbreviations

4OHT: 4-hydroxytamoxifen
ATC: Anaplastic thyroid carcinoma
BrdU: Bromodeoxyuridine;
CM: Conditioned medium
CSF-1: Colony-stimulating factor 1
DMEM: Dulbecco’s Modified Eagle’s Medium
ELISA: Enzyme Linked Immunosorbent Assay
FACS: Fluorescence-activated cell sorting
FBS: Fetal Bovine Serum
FTC: Follicular thyroid carcinoma
hrM-CSF: human recombinant macrophage colony-stimulating factor
LPS: Lipopolysaccharides
OIS: Oncogene-induced senescence
PBS: Phosphate-buffered saline
PCR: Polymerase Chain Reaction
PDTC: Poorly differentiated thyroid carcinoma
PTC: Papillary thyroid carcinoma
PTh: Proliferating thyrocytes
PTh-Macro: Proliferating thyrocytes conditioned macrophages
PTMC: Papillary thyroid microcarcinoma
RPMI: Roswell Park Memorial Institute
SASP: Senescence Associated Secretory Phenotype
STh: Senescent thyrocytes
STh-Macro: Senescent thyrocytes conditioned macrophages
STR: Short Tandem Repeat
TAMs: Tumor associated macrophages
TCV: Tall cell variant
TuC: Thyroid carcinoma cell lines
TuC-Macro: Thyroid carcinoma cell lines conditioned macrophages
