# Supplementary material for: Senescent thyrocytes and thyroid tumor cells induce M2-like macrophage polarization of human monocytes via a PGE2-dependent mechanism
Source: J Exp Clin Cancer Res. 2019 May 21;38:208. doi: 10.1186/s13046-019-1198-8 (PMC6528237; doi:10.1186/s13046-019-1198-8)
Supplement: Supplementary file 1 — Supplementary Results and Methods. (DOCX 16 kb) [file 13046_2019_1198_MOESM1_ESM.docx]

**Additional file 1**

**SUPPLEMENTARY RESULTS**

**Characterization of *in vitro* model of thyrocyte senescence.** To better characterize our senescent thyrocyte model, we investigated the effect of short 4OHT treatment. First, we analyzed ER:RAS thyrocytes treated with 4OHT for 4 days. As shown in Additional file2: Figure S1a, 4 days 4OHT treatment was sufficient to induce morphology changes, increase of p16INK4a expression, and reduction of BrdU incorporation. We next evaluated if acute 4OHT stimulation was sufficient to promote the senescence program. ER:RAS thyrocytes were treated with 4OHT for 24 or 48 hours, and analyzed 7 and 14 days later. In cells treated for 24 hours, a robust induction of ER:RAS protein expression was detected at day 7, which was associated with increase of p16INK4a, reduction of BrdU incorporation (Additional file2: Figure S1b) and changes in cell morphology (data not shown). In cells treated for 48 hours, ER:RAS protein induction was marked at 7 days and significantly reduced at 14 days. Nevertheless, at both time points, a marked increase in the expression of p16INK4a, a stark reduction of BrdU incorporation (Additional file2: Figure S1b) and changes in cell morphology (data not shown) were detected. These results suggest that ER:RAS protein expression obtained by an acute exposure to 4OHT may be sufficient to trigger senescence in our model.

Furthermore, we assessed the minimum effective 4OHT dose able to induce senescence. We treated ER:RAS thyrocytes with different concentration of 4OHT (range 1 - 200 nM) for 5 days, and then evaluating the presence of senescence-specific markers (Additional file2: Figure S1c). A dose-dependent ER:RAS protein expression was detected. The increasing of p16INK4a expression was already evident in 1 nM 4OHT treated cells; the inhibition of proliferation was modest in 1 nM 4OHT treated cells, and more pronounced at higher doses. These results indicate that 5 nM 4OHT may be sufficient to trigger the senescence program consequent to ER:RAS protein expression.

**SUPPLEMENTARY METHODS**

**Cell proliferation assay**

Cells were plated in 96-well cell-culture plates. Proliferation was determined using the Cell Proliferation ELISA kit, BrdU (11669915001, Roche, Basel, Switzerland) following the manufacturer’s instructions.

**Western blotting analysis**

Western blot analysis was performed as previously described (1), using the following antibodies: p16INK4a (BD Biosciences, Milan, Italy); β-actin (Sigma Aldrich, St Louis, MO, USA); anti-Pan-Ras (Calbiochem, San Diego, CA, United States). Immunoreactive bands were visualized using horseradish peroxidase-conjugated secondary antibodies followed by enhanced chemiluminescence (GE Healthcare, Buckinghamshire, UK).

(1) Vizioli MG, Santos J, Pilotti S, Mazzoni M, Anania MC, Miranda C, et al. Oncogenic RAS-induced senescence in human primary thyrocytes: molecular effectors and inflammatory secretome involved. Oncotarget 2014 Sep 30;5(18):8270-83.
