# Supplementary figures and images for: Senescent thyrocytes and thyroid tumor cells induce M2-like macrophage polarization of human monocytes via a PGE2-dependent mechanism
Source: J Exp Clin Cancer Res. 2019 May 21;38:208. doi: 10.1186/s13046-019-1198-8 (PMC6528237; doi:10.1186/s13046-019-1198-8)

**a**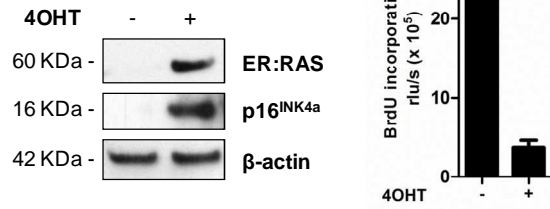**b**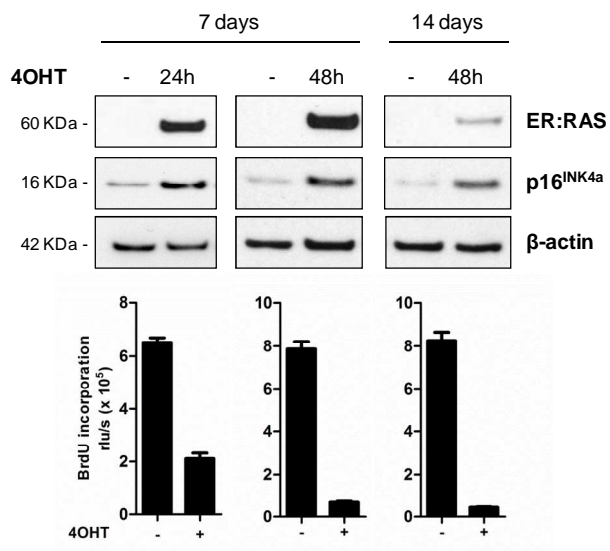**c**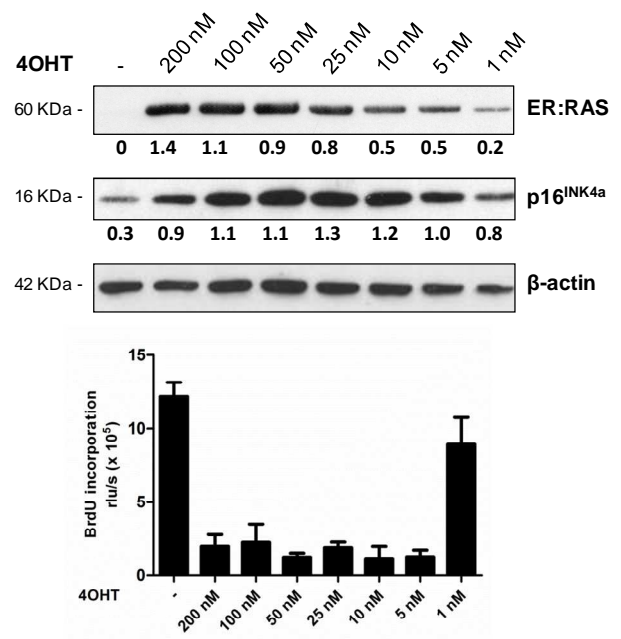

Supplement: Supplementary file 2 — Figure S1. Detection of senescence markers in ER:RAS human primary thyrocytes untreated or treated with 4OHT. Cells were analyzed by WB for the expression of ER:RAS and p16INK4a proteins (β-actin represents loading control), and by BrdU incorporation assay for cell proliferation. Cells were treated with 4OHT for 4 days (a); for 24 or 48 h and monitored for the presence of senescence features at 7 or 14 days (b). In (c), the determination of the minimum 4OHT dose capable to induce thyrocyte senescence was assessed; in WB, values represent band densitometric analysis, normalized to β-actin loading control. For all BrdU experiments, bars represent mean + the standard deviation of five technical replicates RLU: relative luminescence unit (PDF 180 kb) [file 13046_2019_1198_MOESM2_ESM.pdf]

**a**

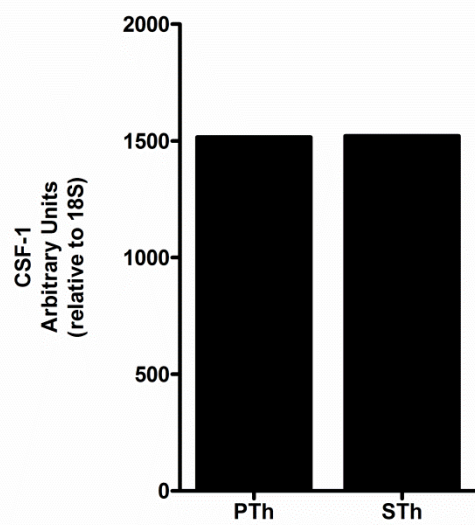

**b**

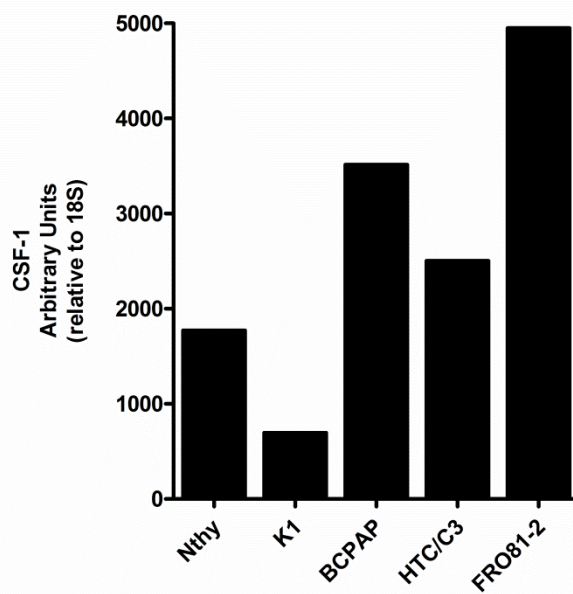

Supplement: Supplementary file 3 — Figure S2. qRT-PCR analysis of CSF-1 transcript levels normalized with the 18S RNA levels in: (a) proliferating (PTh) and senescent (STh) thyrocytes; (b) normal thyroid cell (Nthy) and tumor thyroid cell lines (K1, BCPAP, HTC/C3 and FRO81–2). (PDF 60 kb) [file 13046_2019_1198_MOESM3_ESM.pdf]

**a**

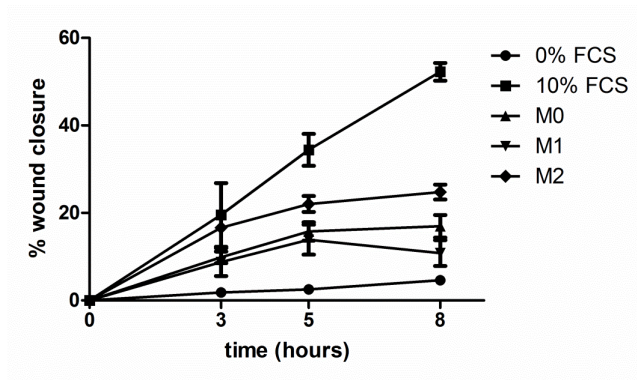

**b**

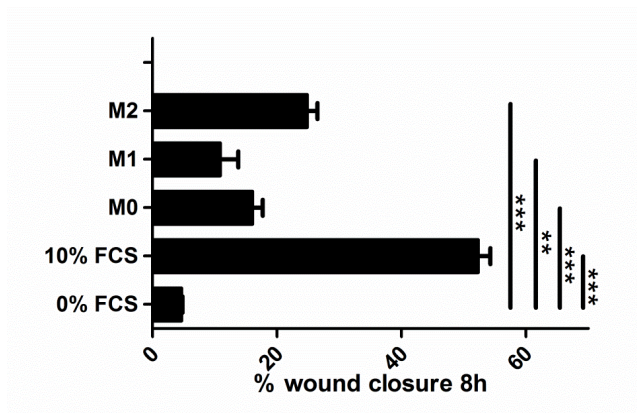

Supplement: Supplementary file 4 — Figure S3. Wound healing assay performed on K1 cells treated with conditioned media from M0, M1 and M2 control macrophages, or with media containing 0% or 10% FCS. The graph shows the percentage of wound closure quantified at the indicated time points (a) and specifically 8 h post-wound (b). Error bars represent standard deviation of four independent experiments. Statistical significance was determined by unpaired t test. ** p < 0.01, ***p < 0.001. (PDF 126 kb) [file 13046_2019_1198_MOESM4_ESM.pdf]

a

## PGE2

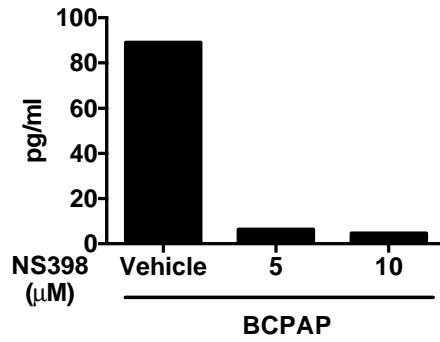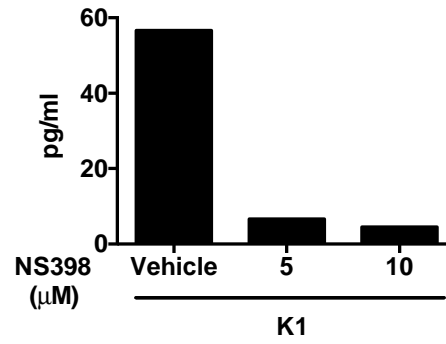

b

## CD206

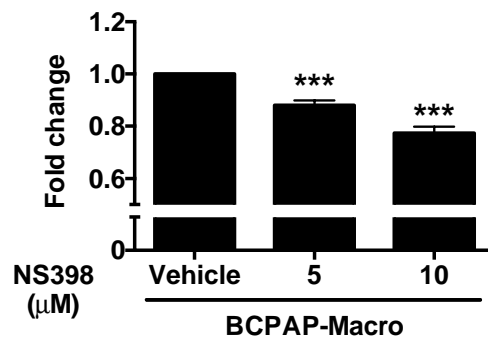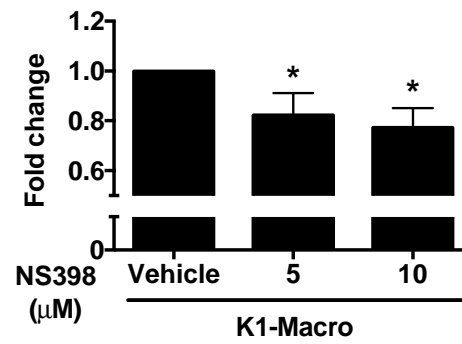

## CCL17

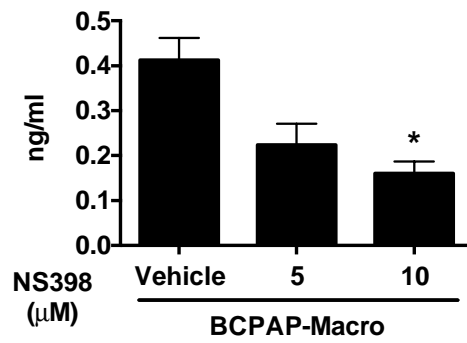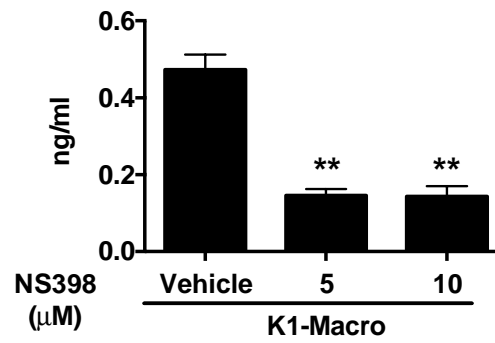

Supplement: Supplementary file 5 — Figure S4. NS398 inhibits PGE2 production and M2-like polarizing ability of BCPAP and K1 thyroid cells. (a) PGE2 secretion, determined by ELISA, of BCPAP and K1 cells treated with NS398 at the indicated doses. (b) Macrophages induced by CM from samples in (a) were analyzed for CD206 expression by FACS and for the secretion of CCL17 by ELISA. Statistical significance was determined by unpaired t test. *p < 0.05, **p < 0.01, ***p < 0.001. (PDF 10 kb) [file 13046_2019_1198_MOESM5_ESM.pdf]

Public dataset from TCGA-Thyroid Carcinoma

**a**

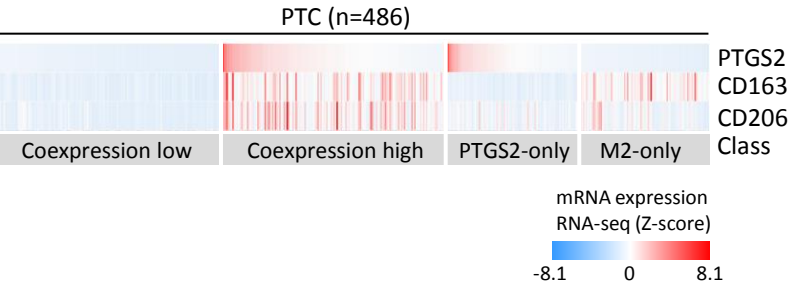

**b**

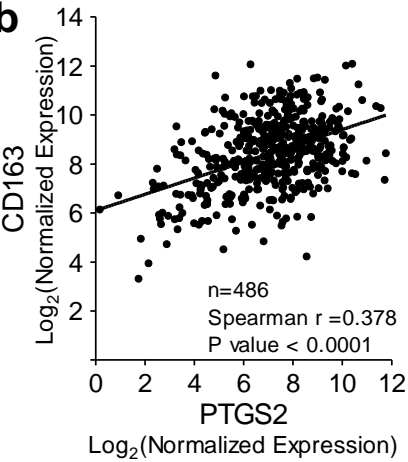

Supplement: Supplementary file 6 — Figure S5. PTGS2 and M2 markers expression in PTCs from TCGA dataset. (a) Heatmap showing PTGS2 and M2 markers (CD163 and CD206) expression across 486 PTCs from TCGA study. Normalized RNA sequencing data of the three genes were downloaded from cBioPortal for Cancer Genomics (www.cbioportal.org; accessed January 2019). Class was established based on PTGS2 and CD163 median expression as described in Fig. 7. (b) Correlation by Spearman coefficient between PTGS2 and CD163 expression in the same cohort. (PDF 208 kb) [file 13046_2019_1198_MOESM6_ESM.pdf]
